# Simple models vs. deep learning in detecting low ejection fraction from the electrocardiogram

**DOI:** 10.1093/ehjdh/ztae034

**Published:** 2024-04-25

**Authors:** John Weston Hughes, Sulaiman Somani, Pierre Elias, James Tooley, Albert J Rogers, Timothy Poterucha, Christopher M Haggerty, Michael Salerno, David Ouyang, Euan Ashley, James Zou, Marco V Perez

**Affiliations:** Department of Computer Science, Stanford University, 353 Jane Stanford Way, Stanford, CA 94305, USA; Department of Medicine, Stanford University, 1265 Pasteur Dr, Stanford, CA 94305, USA; Department of Medicine, Columbia University Irving Medical Center, 622 W 168th St, New York, NY 10032, USA; Department of Medicine, Stanford University, 1265 Pasteur Dr, Stanford, CA 94305, USA; Department of Medicine, Stanford University, 1265 Pasteur Dr, Stanford, CA 94305, USA; Department of Medicine, Columbia University Irving Medical Center, 622 W 168th St, New York, NY 10032, USA; Department of Medicine, Columbia University Irving Medical Center, 622 W 168th St, New York, NY 10032, USA; Department of Medicine, Stanford University, 1265 Pasteur Dr, Stanford, CA 94305, USA; Cedars-Sinai Medical Center, Department of Cardiology, Smidt Heart Institute, 127 S San Vicente Blvd Pavilion, Suite A3600, Los Angeles, CA 90048, USA; Department of Medicine, Stanford University, 1265 Pasteur Dr, Stanford, CA 94305, USA; Department of Biomedical Data Science, Stanford University, 1265 Welch Road, Stanford, CA 94305, USA; Department of Medicine, Stanford University, 1265 Pasteur Dr, Stanford, CA 94305, USA

**Keywords:** Artificial intelligence, Electrocardiograms, Deep learning, Explainability, Interpretability

## Abstract

**Aims:**

Deep learning methods have recently gained success in detecting left ventricular systolic dysfunction (LVSD) from electrocardiogram (ECG) waveforms. Despite their high level of accuracy, they are difficult to interpret and deploy broadly in the clinical setting. In this study, we set out to determine whether simpler models based on standard ECG measurements could detect LVSD with similar accuracy to that of deep learning models.

**Methods and results:**

Using an observational data set of 40 994 matched 12-lead ECGs and transthoracic echocardiograms, we trained a range of models with increasing complexity to detect LVSD based on ECG waveforms and derived measurements. The training data were acquired from the Stanford University Medical Center. External validation data were acquired from the Columbia Medical Center and the UK Biobank. The Stanford data set consisted of 40 994 matched ECGs and echocardiograms, of which 9.72% had LVSD. A random forest model using 555 discrete, automated measurements achieved an area under the receiver operator characteristic curve (AUC) of 0.92 (0.91–0.93), similar to a deep learning waveform model with an AUC of 0.94 (0.93–0.94). A logistic regression model based on five measurements achieved high performance [AUC of 0.86 (0.85–0.87)], close to a deep learning model and better than N-terminal prohormone brain natriuretic peptide (NT-proBNP). Finally, we found that simpler models were more portable across sites, with experiments at two independent, external sites.

**Conclusion:**

Our study demonstrates the value of simple electrocardiographic models that perform nearly as well as deep learning models, while being much easier to implement and interpret.

## Introduction

Left ventricular systolic dysfunction (LVSD) is a characteristic feature of patients with heart failure, but it can be challenging to screen for.^1^ At the two ends of the complexity spectrum, the N-terminal prohormone brain natriuretic peptide (NT-proBNP) laboratory biomarker is an inexpensive screening tool for heart failure but suffers from modest performance,^2,3^ while the transthoracic echocardiogram (TTE) provides a gold-standard diagnosis for LVSD but is expensive and time-consuming relative to other tests.^4^ An ideal screening tool would fall somewhere in the middle by being inexpensive, while offering high accuracy. One such candidate is electrocardiogram (ECG)-based risk scores. Historically, no reliable method existed to detect reduced ejection fraction from the ECG waveform,^5^ but recently, deep learning methods have demonstrated impressive performance.^6^ The same trend is true for other important tasks, including detecting atrial fibrillation in sinus rhythm,^7^ detecting valvular disease,^8^ and predicting future mortality.^9,10^

While highly performant, deep learning has several key limitations, including vulnerability to domain shifts across hospitals,^11^ imaging vendors^12^ or demographic groups,^13^ lack of interpretability,^14^ and challenges around implementation.^15–17^ Whether simpler, non-deep learning methods can offer similar performance on complex ECG tasks remains unclear. Historically, ECG-based diagnostic tools for identifying less complex conditions took the form of simple criteria,^18,19^ decision trees,^20^ and linear models^21^ based on hand-measurable features such as the PR interval and the amplitude of the T wave. In these simpler cases, deep learning offers only slight gains over simpler criteria applied by humans.^22^ Recently, a large data set of simple measurements was mined to build a simple model to detect atrial fibrillation in sinus rhythm, although this model far under-performed deep learning methods,^7,23^ and at least one other deep learning model was presented alongside a strong decision tree–based baseline based on automated measurements.^9^

In this study, we set out to understand how simpler models based on automated ECG measurements compare with deep learning models in detecting LVSD from the ECG. Using a data set of matched 12-lead ECGs and TTEs from the Stanford University Medical Center, we trained a range of models with increasing complexity to detect low ejection fraction from the ECG. We found that a random forest model on 555 discrete, automated measurements performed similarly to deep learning methods, and a linear model based on five automated measurements performed only slightly worse than deep learning but better than NT-proBNP. This continuum of models, trading off complexity and accuracy, demonstrates that simpler methods can sometimes be substituted for deep learning models, allowing for greater interpretability and ease of implementation.

## Methods

### Study populations and data sources

We trained and primarily evaluated a range of simple-measurement-based and deep learning models to detect LVSD, defined as a left ventricular ejection fraction (LVEF) below 35%, from ECGs. Models were trained using a data set of paired 12-lead resting ECGs and TTEs from the Stanford University Medical Center. This data set consisted of all TTEs that were taken during the course of clinical care between March 2008 and May 2018 with an ECG within 2 weeks. Electrocardiograms that did not pass the Phillips TraceMaster quality control standard were removed. All ECGs falling within 2 weeks of a TTE were included, with a label corresponding to the closest TTE; in other words, multiple ECGs could be assigned to a single TTE, but only the closest TTE was assigned to a given ECG. In the test set, only the first ECG was used per patient after this pairing. We extracted 39 019 TTE–ECG pairs from 27 763 patients, which were then split by patient into train, validation, and test sets in a 5:1:4 ratio.

In the test set, we included only the first ECG per patient (*Figure 2*). These ECGs were saved as 10 s signals from all 12 leads of the ECG, sampled at 500 Hz. We extracted ECG waveforms at 250 Hz, along with measurements and text over-reads from TraceMaster. We included all 555 measurements that had numerical values pertaining to a waveform structure.

Left ventricular ejection fractions were extracted from STARR-OMOP,^24^ a common data model of Stanford electronic health records, based on echocardiograms acquired using iE33, Sonos, Acuson SC2000, Epiq 5G, or Epiq 7C ultrasound machines and interpreted by a cardiologist during standard clinical practice. We included all measurements within 2 weeks of recording an echo procedure. We defined LVSD as an LVEF below 35%. We also extracted NT-proBNP from STARR-OMOP and included all records within 30 days of the reference ECG.

A data set from the Columbia Irving Medical Center was used as a first external validation cohort. The Columbia data set was constructed similarly to that of Stanford, but a different ECG vendor (the General Electric MUSE system) was utilized. After inclusion criteria were applied, a random subsample of data was included for analysis. We additionally used a second external data set from the UK Biobank. This cohort was substantially different from the first two hospital-based data sets, being made up of a cross section of mostly healthy British patients. In the UK Biobank, all patients with a 12-lead resting ECG and cardiac magnetic resonance imaging (cMRI) taken at the first imaging visit were included. All paired ECG and cMRI studies took place on the same day; details of the cMRI protocol are available in the literature.^25^ A previously described deep learning pipeline^26^ was used to estimate the LVEF from the cMRI. Other ECG abnormalities were determined based on the ECG text over-read using string matching, validated by manual inspection. The UK Biobank ECGs were also recorded using General Electric ECG machines.

### Model development and training

Deep learning models were trained using Python 3.9 and PyTorch 1.11 on single Nvidia Titan Xp GPUs using Stanford’s Sherlock computing cluster. We closely followed the architecture described in previous literature for detecting LVSD^6^ and found that exploring different architectures did not provide a significant increase in validation area under the receiver operator characteristic curve (AUC). To evaluate deep learning models at other sites, we ran the model on data using a range of pre-processing parameters and reported the best performance, since different sites and vendors may use different pre-processing parameters and follow different types of distribution. We evaluated the deep learning model on eight different pre-processing set-ups at each external validation site and reported results on the best-performing one. The set-ups included processing signals with and without band-pass filters, with and without wandering baseline filters, and with and without per-lead normalization to mean 0 and standard deviation 1. (Stanford data were prepared with all three of these.) In our previous experience, this had proved sufficient to allow models to perform well across sites. Random forest models were trained using Python 3.9 and XGBoost 1.7, employing the binary logistic loss. We trained several models with different tree depths and numbers of trees using grid search and selected the best model based on validation AUC. Linear models were trained using Python 3.9 and Scikit-Learn 1.2 with standard logistic regression, without regularization or normalization unless otherwise mentioned. All analyses were performed by training models on the training set and selecting variables, hyperparameters, and models based on results in the validation set. After the models were finalized, their performance was evaluated on the test set and external validation sets. To select a shortlist of variables for smaller models, we selected a list of variables familiar to clinicians based on inspection and iteratively fit increasingly lasso-regularized models, while removing correlated variables, and then trained an un-regularized model on those variables to achieve maximal performance. We selected a model of a size where removing any one variable would cause a drop in performance of >1% in the validation set, while adding any one variable would cause an improvement in performance of <1%.

### Statistical analysis

We primarily compared models based on the AUC, a standard metric used for evaluating a predictor’s performance across multiple cut-offs in binary classification tasks. All AUCs were computed using the Scikit-Learn Python package. We additionally computed sensitivity, specificity, and positive predictive values using standard definitions. We reported balanced sensitivity and specificity (choosing the cut-off that minimizes the difference between sensitivity and specificity), positive predictive value at the same cut-off, sensitivity at 90% specificity, and specificity at 90% sensitivity. All confidence intervals were 95% intervals generated through bootstrapping with 1000 samples.

## Results

### Study population

We trained several models on ECGs paired with TTEs from the Stanford University Medical Center taken between March 2008 and May 2018 during the normal course of clinical practice (*Figure 2*). From the 96 361 resting TTEs (from 54 045 patients) with a recorded ejection fraction, 46 254 (32 361 patients) occurred within 2 weeks of a unique ECG. Among those, 40 994 ECGs (28 949 patients) passed the automated quality control test performed by using the Philips TraceMaster software, while 5260 ECGs (3412 patients) were removed. We randomized those pairs by patient 50%/10%/40% into train, validation, and test sets, resulting in 20 269 training ECG–TTE pairs (14 448 patients), 4276 validation ECG–TTE pairs (2983 patients), and 16 449 ECG–TTE pairs (11 518 patients) randomized to the test group, of which 11 518 first ECGs per patient were included in the test set. In the train, validation, and test sets, 2175 (10.73%), 462 (10.80%), and 1119 (9.72%) ECGs, respectively, were taken from patients with LVSD (in the test set, this was also the number of patients). Detailed demographic data are presented in *Table 1*.

**Table 1:** Demographics in each split

	Train	Valid	Test	Test, BNP subcohort	UK Biobank	Columbia
Count	20 269	4276	11 518	2163	34 280	36 975
LVSD	2175 (10.73%)	462 (10.80%)	1119 (9.72%)	476 (22.01%)	96 (0.28%)	4656 (12.59%)
LVEF	55.35 (13.31)	55.36 (13.36)	56.25 (13.21)	50.11 (16.71)	59.57 (6.16)	53.85 (13.49)
Age	61.5 (18.0)	61.7 (17.5)	62.1 (17.6)	65.3 (17.1)	63.6 (7.6)	64.0 (16.5)
Male gender	11 533 (56.90%)	2385 (55.78%)	6358 (55.20%)	1235 (57.10%)	16 396 (47.83%)	19 645 (53.13%)
Female gender	8735 (43.10%)	1891 (44.22%)	5160 (44.80%)	928 (42.90%)	17 884 (52.17%)	17 319 (46.84%)
Other/unknown gender	1 (0.00%)	0 (0.00%)	0 (0.00%)	0 (0.00%)	0 (0.00%)	11 (0.03%)
White	11 427 (56.38%)	2492 (58.28%)	6500 (56.43%)	1164 (53.81%)	33 166 (96.75%)	14 106 (38.15%)
Asian	2917 (14.39%)	586 (13.70%)	1743 (15.13%)	318 (14.70%)	459 (1.34%)	767 (2.07%)
Black or African American	1089 (5.37%)	258 (6.03%)	590 (5.12%)	109 (5.04%)	226 (0.66%)	5781 (15.63%)
Other/unknown race	4836 (23.86%)	940 (21.98%)	2685 (23.31%)	572 (26.44%)	429 (1.25%)	16 321 (44.14%)
Hispanic	2647 (13.06%)	539 (12.61%)	1374 (11.93%)	300 (13.87%)		9623 (26.03%)
Non-Hispanic	17 622 (86.94%)	3737 (87.39%)	10 144 (88.07%)	1863 (86.13%)		16 105 (43.56%)
Unknown ethnicity	0 (0.00%)	0 (0.00%)	0 (0.00%)	0 (0.00%)		11 247 (30.42%)
Sinus rhythm	15 587 (76.90%)	3298 (77.13%)	8940 (77.62%)	1564 (72.31%)	33 563 (97.91%)	
Pacemaker	1664 (8.21%)	330 (7.72%)	745 (6.47%)	217 (10.03%)	55 (0.16%)	
Premature ventricular complexes	1367 (6.74%)	287 (6.71%)	757 (6.57%)	196 (9.06%)	1201 (3.50%)	
Left bundle branch block	975 (4.81%)	224 (5.24%)	535 (4.64%)	142 (6.56%)	311 (0.91%)	

Blank entries are missing: Hispanic ethnicity was not tracked in the UK Biobank, race and ethnicity were not tracked for all patients in the Columbia cohort, and ECG findings were not available in the Columbia cohort.

To understand how portable models performed across sites, we additionally evaluated our models on ECGs from another healthcare system, the Columbia Irving Medical Center, and a prospective population of healthy individuals, the UK Biobank cohort. The Columbia cohort consisted of 36 975 patients who received an ECG and TTE at the Columbia Medical Center within a 2-week window. In that group, prevalence was similar to that at Stanford (12.59%), and there were greater proportions of Black and Hispanic patients (*Table 1*). The UK Biobank cohort consisted of 34 280 patients from the general population who prospectively received cMRI, and had a much lower prevalence rate of LVSD, with just 96 (0.28%) cases.

The population also had higher rates of normal ECGs (97.9% in the UK Biobank vs. 77.6% at Stanford) and contained a greater proportion of White patients (96.75% in the UK Biobank vs. 56.38% at Stanford).

### Simple models using discrete, automated measurements detect left ventricular systolic dysfunction almost as well as deep learning models

**Figure 1:**
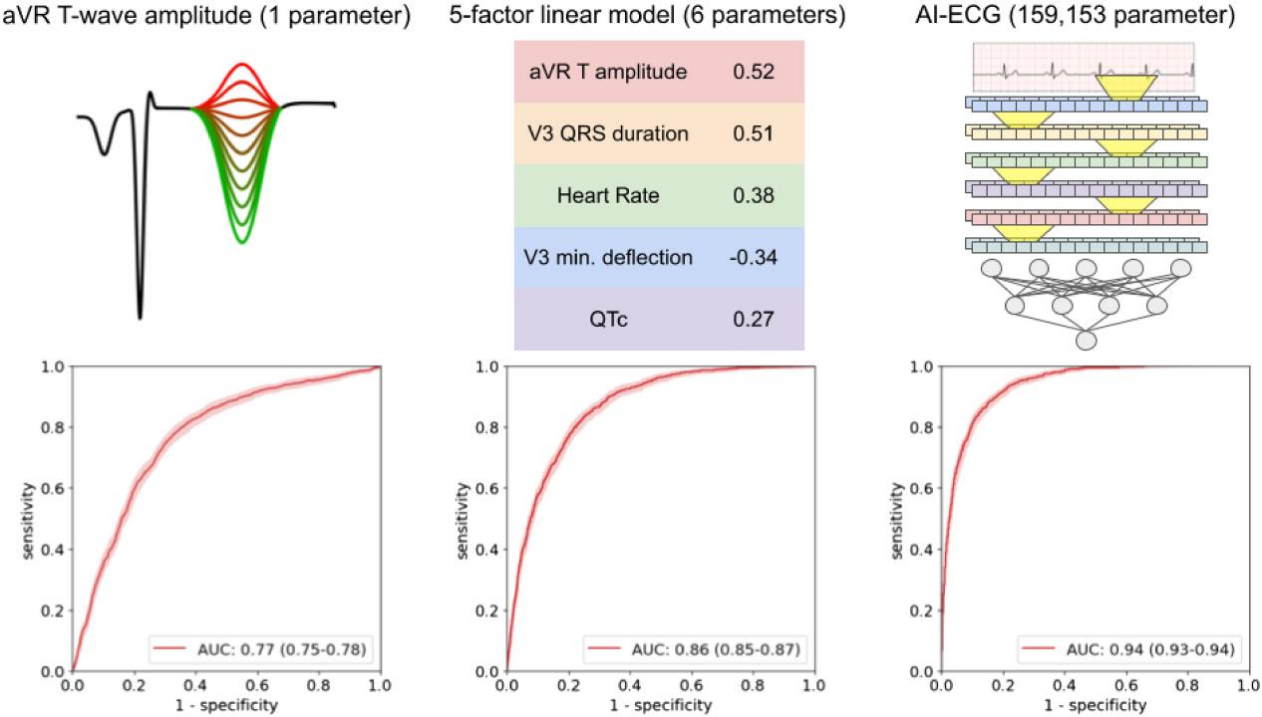
Receiver operator characteristic curves for three risk scores for detecting left ventricular systolic dysfunction. Left: the amplitude of the T wave in Lead aVR, used directly as a risk score for left ventricular systolic dysfunction. Centre: a linear model based on five electrocardiogram measurements. Weights based on normalized measurements are shown. Right: a deep learning model based on the electrocardiogram waveform (diagram is a simplification for illustrative purposes only).

**Figure 2:**
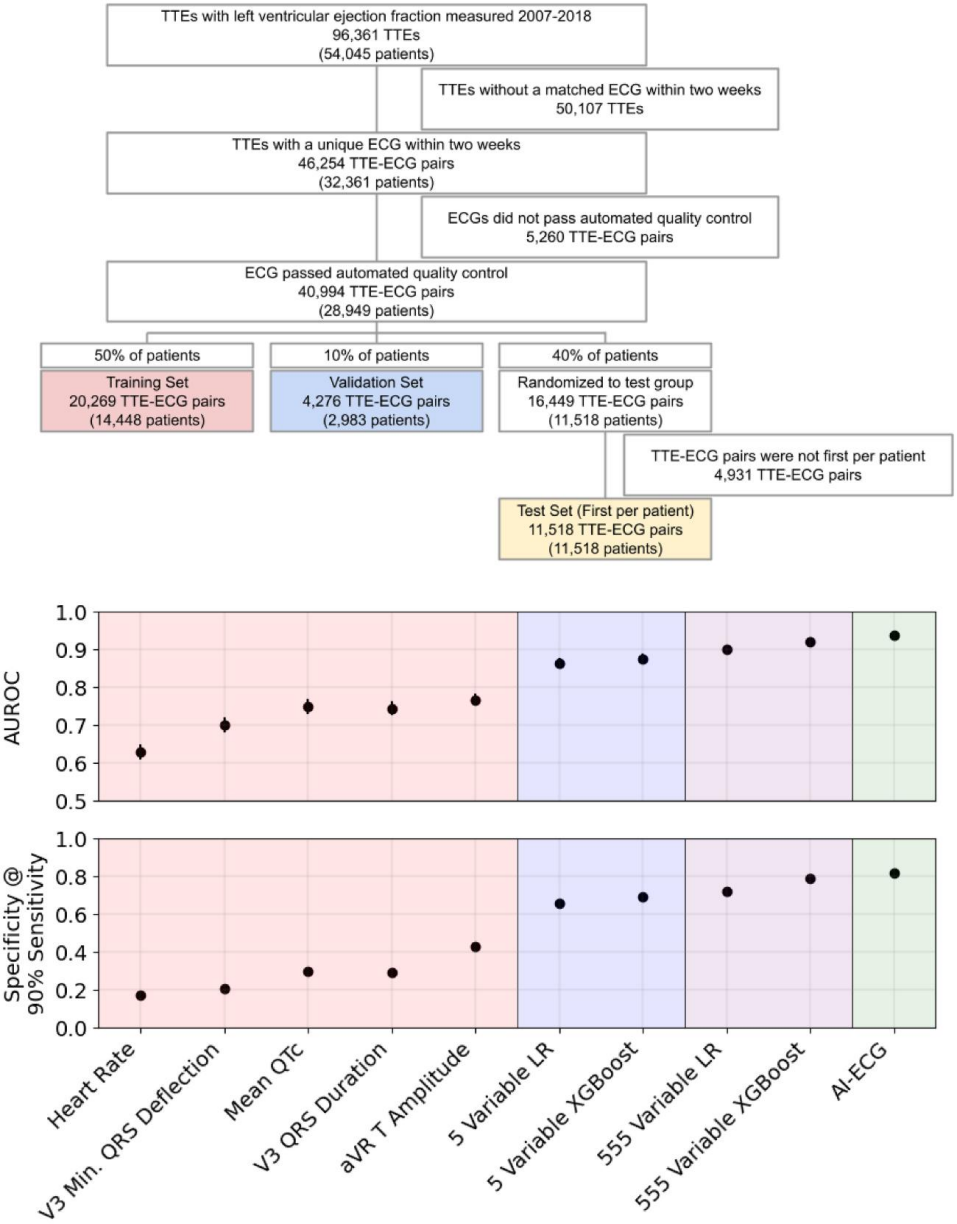
Top panel: a consort diagram for the Stanford cohort. Bottom panel: the performance of several risk scores in detecting left ventricular systolic dysfunction, by the area under receiver operator characteristic curve and specificity at a cut-off providing 90% sensitivity. The error bars are 95% bootstrap confidence intervals.

The convolutional neural network trained on 12-lead ECG waveforms achieved an AUC of 0.94 (0.93–0.94) in detecting LVSD, comparable to the 0.93 previously reported^6^ (*Figures 1* and *2*; previous work did not report a confidence interval on the computed AUC). Choosing a cut-off to balance sensitivity and specificity resulted in values of 0.86 (0.84–0.88) and 0.86 (0.86–0.87), respectively. At that cut-off, it achieved a positive predictive value of 0.40 (0.37–0.42). At a sensitivity rate of 90%, it achieved a specificity of 0.82 (0.81–0.83). The model consisted of 159 153 trainable parameters.

To understand how well discrete ECG measurements can be used to detect LVSD, we next trained lasso and random forest models to detect LVSD based on 555 ECG measurements extracted by the Philips TraceMaster software (listed in Supplementary material online, *Table S2*). Examples of such measurements (in order of increasing complexity) are the heart rate, the P-wave amplitude in Lead I, the area under the QRS complex in Lead aVL, and the maximal T-wave angle through the transverse plane. The random forest achieved an AUC of 0.92 (0.91–0.93), not significantly different from that of the deep learning model (*P* = 0.08). The best-performing random forest consisted of 50 trees of depth 7, resulting in 6350 binary cut-offs. The lasso linear model achieved an AUC of 0.90 (0.89–0.91), using only 556 trainable parameters. The weights of the linear model are shown in Supplementary material online, *Table S2*. The performance of the models based on one to eight measurements is shown in Supplementary material online, *Figure S2*.

Acknowledging that a 555-measurement linear model is still not easily ‘interpretable’, we reduced the number of measurements further, first limiting the list to familiar measurements and then using lasso regression and removing correlated features. We arrived at a shortlist of five measurements that can be easily manually assessed in a clinical setting (*Table 2*): the T-wave amplitude in aVR; the QRS duration in V3; the mean QTc (corrected QT interval using Bazett’s formula); the maximum negative QRS deflection in V3 (the greater of the Q and S amplitudes); and the heart rate. In all cases, the correlation was positive, except for the maximum negative QRS deflection in V3 (i.e. a deeper Q or S wave in V3 indicates greater risk, while a shallower or positively inverted T wave in aVR indicates greater risk). A random forest trained on these five measurements achieved an AUC of 0.88 (0.87–0.89), while a linear (non-lasso) model achieved an AUC of 0.86 (0.85–0.87). A ‘quadratic’ model consisting of a linear combination of each measurement, its square, and the product of each pair of measurements, did not perform better than the linear model, with an AUC of 0.85 (0.84–0.86). The random forest consisted of 20 trees of depth 4, or 80 total parameters, while the linear model used 6 trainable parameters, each one easily interpretable. Notably, the linear model fared only slightly worse than the deep learning model while using only 6 vs. 159 153 trainable parameters. In a subcohort of 2097 patients in the test set who received an NT-proBNP laboratory test within 30 days of the reference ECG, the linear model modestly outperformed that test, achieving an AUC of 0.79 (0.77–0.80) vs. 0.77 (0.75–0.79). Specificity at 90% sensitivity followed a similar trend to AUC (*Figure 2*; Supplementary material online, *Table S2*).

**Table 2:** A logistic regression model for detecting left ventricular systolic dysfunction

	Units	Coefficient (absolute)	Coefficient (normalized)	*P*-value	Independent AUC
aVR T amplitude	µV	3.69E−3 (3.35E−3 to 4.04E−3)	0.52 (0.47–0.57)	7.22E−96	0.77 (0.75–0.78)
V3 QRS duration	ms	2.03E−2 (1.82E−2 to 2.23E−2)	0.51 (0.45–0.56)	2.56E−81	0.75 (0.73–0.76)
Heart rate	b.p.m.	1.97E−2 (1.73E−2 to 2.21E−2)	0.38 (0.34–0.43)	1.17E−58	0.63 (0.61–0.65)
V3 QRS minimum deflection	µV	−4.76E−4 (−5.34E−4 to −4.18E−4)	−0.34 (−0.38 to −0.30)	7.07E−58	0.70 (0.68–0.72)
QTc	ms	6.73E−3 (5.38E−3 to 8.08E−3)	0.27 (0.22–0.33)	1.50E−22	0.75 (0.73–0.77)
Intercept	Unitless	−9.10 (−9.66 to −8.55)	−2.61 (−2.68 to −2.55)	3.11E−225	

Coefficients for absolute covariates and covariates normalized to mean 0 and standard deviation 1 are shown, along with units for each absolute covariate, *P*-values for each coefficient and AUCs for each covariate as an independent predictor.

### Single electrocardiogram measurements detect left ventricular systolic dysfunction with high accuracy

For each of the 555 numerical measurements taken by using the Philips TraceMaster software, we calculated the AUC when using the measurement as an independent predictor of LVSD (see Supplementary material online, *Table S2*). For each of the five measurements used in the small linear model, we evaluated their independent performance in detecting LVSD in the test set (*Figure 2*). The best-performing measurement was the T-wave amplitude in aVR, which independently achieved an AUC of 0.77 (0.76–0.78). The QRS durations in V3 and mean QTc were also high, achieving AUCs of 0.75 (0.73–0.76) and 0.75 (0.74–0.77), respectively, while the V3 Min. The QRS deflection achieved 0.70 (0.68–0.72) and heart rate 0.63 (0.61–0.65).

### Simpler models perform better across sites

To understand the portability of simple and deep learning models across sites, we evaluated our models in two external cohorts, the UK Biobank cohort and Columbia cohorts. The deep learning model did not perform as well on UK Biobank data, with an AUC of 0.74 (0.69–0.78; *Table 3*), but achieved good performance in the Columbia cohort, with an AUC of 0.88 (0.87–0.88). The large difference in the UK Biobank cohort may be due to subtle differences in vendor waveform pre-processing, although we were unable to detect any differences through inspection (differences in the population or the use of cMRI vs. echocardiogram are also a plausible explanation, but are mostly ruled out by the following results). The simpler, measurement-based models, on the other hand, performed similarly to Stanford: the linear model achieved AUCs of 0.83 (0.78–0.87) and 0.80 (0.80–0.81) in the UK Biobank and Columbia cohorts, respectively, vs. 0.86 at Stanford, and the random forest model achieved AUCs of 0.82 (0.77–0.87) and 0.81 (0.80–0.82), respectively, vs. 0.88 at Stanford, demonstrating portability to radically different populations like the one in the UK Biobank cohort. The T-wave amplitude in aVR achieved similar performance in both the UK Biobank, with an AUC of 0.78 (0.73–0.83), and Columbia, with an AUC of 0.74 (0.74–0.75). Other individual measurements were similarly predictive in the UK Biobank and Columbia data sets (see Supplementary material online, *Figure S2*). Due to a lack of available measurements, we were unable to evaluate the 555-measurement models at external sites.

**Table 3:** Area under the receiver operator characteristic curves of different predictors/models for left ventricular systolic dysfunction across multiple sites

	aVR T amplitude	5‐Measurement LR	5‐Measurement XGBoost	AI-ECG
Stanford	0.77 (0.75–0.78)	0.86 (0.85–0.87)	0.88 (0.87–0.89)	0.94 (0.93–0.94)
UKB	0.78 (0.73–0.83)	0.83 (0.78–0.87)	0.82 (0.77–0.87)	0.72 (0.67–0.78)
Columbia	0.74 (0.74–0.75)	0.80 (0.80–0.81)	0.81 (0.80–0.82)	0.88 (0.87–0.88)

## Discussion

We found that simple models based on discrete, automated ECG measurements detected LVSD with impressive performance, almost as well as deep learning models using waveforms and much better than standard laboratory tests. The first strength of this study is that it is among the first to use a deep learning model using ECG waveforms, considered the optimal strategy, to benchmark the performance of simpler ECG models, revealing simple strategies that perform nearly as well as the best-performing complex models. The second strength is that it presents tangible tools that could be easier to deploy than those deep learning models. The third is that it demonstrates for the first time that these tools are more portable to different sites and populations.

While in an idealized setting, medical systems would use tools with the highest possible accuracy, using simpler models has a number of benefits with respect to real-world application. As highlighted by our multicentre validation results, models with simpler inputs often exhibit stronger performance when transferred to other sites with different vendors and demographics. They are also easier to troubleshoot, and to detect unintended domain shifts in input data, since the distribution of input measurements is much simpler. These simpler models are also much more interpretable and can provide insight to physicians in ways that deep learning and even more complicated linear and tree-based methods cannot. In this study, we show a continuum of models (*Figures 1* and  *2*) that trade off complexity and performance. Notably, models based on automated measurements have been enabled by the same big data revolution that has enabled deep learning methods; previously, large data sets of automated measurements were not available, and the measurements were not available in real time for inference.

The five-variable linear model that we trained can be directly interpreted and linked to known electro-physiologic consequences of LVSD. The development of LVSD is marked by the progressive accumulation of depolarization and repolarization abnormalities, such as abnormal QRS complexes, delays in repolarization (with prolonged QT), and more prominent T-wave abnormalities, as captured by our model. Elevated heart rate^27^ and prolonged QT interval^28,29^ are both well-known to be related to the severity and prognosis of LVSD. Progressive LVSD leads to decreased stroke volume and an elevated heart rate is frequently a compensatory mechanism to maintain cardiac output in addition to being a marker of atrial arrhythmias that frequently accompany heart failure. The highest weighted measurement in the regression is the T-wave amplitude in aVR, which also independently predicts LVSD with an AUC of 0.77. This measurement was previously shown to be a strong predictor of cardiovascular and all-cause mortality,^30^ despite evidence that clinicians often ignore Lead aVR completely when reading ECGs.^31^ An upward-facing T wave in aVR is also correlated with an ischaemic aetiology of cardiomyopathy.^32^ Deep Q or S waves in V3 are indicative of late QRS transition, which has previously been associated with the risk of sudden cardiac death,^33^ while prolonged QRS complexes are known to be associated with LVSD.^34^ The success of the small models that we present both confirms previous trends in the literature and finds new connections between the ECG and the LVSD, while also providing a new and simple diagnostic tool.

Our work has limitations. While we present strong, simple models for detecting LVSD, they do not perform as well as deep learning models in terms of accuracy, but rather present different points on the continuum between complexity and performance. We evaluated the performance of NT-proBNP as a baseline predictor for LVSD, as this serum biomarker is typically increased in patients with heart failure with reduced ejection fraction (HFrEF). However, it should be noted that this parameter is imperfect for the detection of LVSD, as patients with well-compensated HFrEF may not have elevated NT-proBNP and patients with heart failure with preserved ejection fraction may have elevated NT-proBNP without LVSD. Our conclusions about LVSD probably do not transfer to all phenotypes; for example, in the case of detecting atrial fibrillation in sinus rhythm, previous studies suggest that deep learning achieves a much higher performance.^7,23^ There are also many cases where deep learning is easier to deploy or much more accurate than measurement-based methods, such as when measurements are unavailable or when only a single lead is available, for example, on smartwatches and other mobile devices. In the case of the 555-measurement models, implementation is restricted to settings where this full list of measurements is available, probably only to settings with similar Philips TraceMaster software to that used at Stanford. The performance of the deep learning model in the UK Biobank cohort was poor, and while this in a sense shows the value of simpler models in terms of portability, it was probably due to technical differences in signal processing between ECG machines that we were not able to identify or correct for. However, the signal processing tools that we used were sufficient to allow portability of models to multiple other sites. Finally, our work was limited to the populations that we described, and accuracy might be diminished in different populations, although we had the benefit of working with three diverse populations (two tertiary care centres in the USA and one biobank in the UK).

In this study, we present a set of simple methods to detect LVSD from the ECG, with performances ranging between those of standard laboratory tests and state-of-the art deep learning methods. In many cases, simpler methods with a slightly lower accuracy based on discrete features may be better to deploy than more complicated, uninterpretable methods, and may yield improved insights into the underlying physiology. We believe that there is benefit to presenting results of study techniques along the continuum of complexity, as different healthcare systems may opt for the employment of different models along this continuum based on resources and accessibility. In the setting of ECG interpretation, this is possible, thanks to a wealth of domain knowledge about important ECG measurements.

### Model and code availability

For normalized inputs, the small linear model weights are shown in *Figure 1*, and the large linear model weights are shown in Supplementary material online, *Table S1*. The code to train deep learning models is available at https://github.com/weston100/AI-ECG.

## Supplementary Material

ztae034_Supplementary_Data

## Data Availability

UK Biobank data are available through application. Data from the Stanford and Columbia Irving Medical Centers cannot be shared due to patient privacy constraints.
