# Supplementary material for: Simple models vs. deep learning in detecting low ejection fraction from the electrocardiogram
Source: Eur Heart J Digit Health. 2024 Apr 25;5(4):427–34. doi: 10.1093/ehjdh/ztae034 (PMC11284011; doi:10.1093/ehjdh/ztae034)

Supplemental Tables

Supplemental Table 1

AUC as independent predictor and LR coefficient for all ECG measurements.

	AUC	Coefficients
I_tptparea	0.78	-0.05
I_tarea	0.77	-0.14
I_tamp	0.77	-0.24
aVR_tptparea	0.77	0.05
aVR_tamp	0.77	0.2
V3_qrsarea	0.76	0.01
aVR_tarea	0.76	0.02
meanqtc	0.75	0.25
V4_qrsdur	0.74	-0.29
V5_qrsdur	0.74	-0.8
meanqrsdur	0.74	-0.33
V3_qrsdur	0.73	4.45
V6_tptparea	0.73	0.12
V6_tamp	0.73	-0.18
V4_qrsarea	0.73	0.21
V6_qrsdur	0.72	-1.59
II_qrsdur	0.72	1.47

V6_tarea	0.72	0.08
aVL_tptparea	0.72	0.27
V2_qrsdur	0.72	0.54
III_qrsdur	0.72	0.38
aVL_tarea	0.72	0.23
aVR_qrsppk	0.72	-0.53
II_ramp	0.71	0.36
V4_ramp	0.71	0.86
aVL_tamp	0.71	-0.6
aVF_qrsdur	0.71	0.31
I_qrsdur	0.71	0.86
tfrontaxis	0.71	0.12
aVL_qrsdur	0.71	0.34
fronttmaxangle	0.7	0.08
aVR_qrsdur	0.69	0.28
fronttinitangle	0.69	0.06
II_tptparea	0.69	0.07
I_stend	0.69	0.19
II_tamp	0.69	0
aVL_vat	0.69	0.26
thorizaxis	0.69	0.04
V2_qrsarea	0.69	0

II_tarea	0.69	0.1
V1_qrsdur	0.68	-0.53
V3_ramp	0.68	-0.31
V3_sdur	0.68	-4.87
frontttermangle	0.68	0.03
V3_rdur	0.67	-4.26
V5_tamp	0.67	-0.11
V5_tptparea	0.67	0.06
aVR_stend	0.67	0.66
frontttermmag	0.67	-0.42
V6_stend	0.67	-0.72
V5_ramp	0.67	-1.03
V5_tarea	0.67	-0.04
I_st80	0.67	-0.38
V6_ston	0.67	0.34
sagqrsmaxmag	0.66	0.36
aVL_stend	0.66	-2.22
I_vat	0.66	-0.1
I_ston	0.66	-1.16
aVR_ston	0.66	-0.11
I_stmid	0.66	1.62
frontqrstermmag	0.65	0.26

transqrstermmag	0.65	-0.08
aVF_qrsarea	0.65	0.14
fronttmaxmag	0.65	-0.36
V3_vat	0.65	0.09
II_qrsarea	0.65	-0.16
aVR_tdur	0.65	1.31
V6_st80	0.65	-0.09
meanrrint	0.65	-0.29
V1_tamp	0.65	0.21
transqrsinitangle	0.65	0.18
aVL_st80	0.65	4.06
transqrsmaxangle	0.65	0.13
aVF_ramp	0.65	-0.96
V1_tptparea	0.65	-0.12
V4_sdur	0.65	0.4
V6_stmid	0.65	0.33
I_stslope	0.65	-0.08
sagqrsmaxangle	0.64	0.07
aVL_stslope	0.64	0.32

I_rdur	0.64	-0.65
stfrontaxis	0.64	-0.06
aVR_st80	0.64	-0.45
transqrscwrot	0.64	0.25
V1_tarea	0.64	-0.12
aVL_rdur	0.64	-0.54
sagqrstermmag	0.63	-0.04
aVR_tptpdur	0.63	-1.44
aVR_stmid	0.63	-0.22
lowventrate	0.63	-0.6
II_qrsppk	0.63	-0.67
meanventrate	0.63	0.01
aVL_stmid	0.63	-1.43
I_tdur	0.63	2.54
III_qrsarea	0.63	0.11
aVL_stdur	0.63	-0.09
V6_rdur	0.63	1.4
III_stdur	0.63	0.25
aVF_stdur	0.63	-0.36
V6_stdur	0.63	-0.12
V5_stdur	0.63	-3.98
V1_stdur	0.63	1.7

I_stdur	0.63	0.42
V3_stdur	0.63	0.97
fronttinitmag	0.63	0.45
V4_stdur	0.63	1.41
aVR_stdur	0.63	0.11
II_stdur	0.63	-0.41
V2_stdur	0.63	-0.09
I_ramp	0.63	-0.32
numberofcomplexes	0.62	0.35
highventrate	0.62	0.46
II_ston	0.62	-0.57
sthorizaxis	0.62	-0.01
transtinitangle	0.62	0.06
V2_rdur	0.62	-0.41
V2_ramp	0.62	-0.01
V5_qrsarea	0.62	0.23
meanqtseg	0.61	-0.76
ventratestddev	0.61	-0.27
V1_ston	0.61	-0.24
V3_samp	0.61	0.3
I_qrsppk	0.61	0.36
II_tdur	0.61	-0.16

II_stend	0.61	2.1
i40horizaxis	0.61	0.02
V2_sdur	0.61	-0.19
V5_stend	0.61	0.5
V6_ramp	0.61	-1.18
sagqrsinitmag	0.61	0.1
V1_qrsarea	0.61	0.05
I_tptpdur	0.61	-2.61
sagqrsinitangle	0.61	0.06
III_stslope	0.6	0.49
sagtmaxmag	0.6	-0.06
V1_ppppdur	0.6	-0.01
aVR_stslope	0.6	0.02
V6_vat	0.6	0.02
V5_ston	0.6	-0.91
transpcwrot	0.6	-0.03
aVR_qrsarea	0.6	0.01
II_tptpdur	0.6	0.06
sagptermangle	0.6	-0.12
V1_qdur	0.6	0.57
III_tamp	0.6	-0.07
V6_stslope	0.6	0.34

V2_vat	0.6	0.03
V2_pppparea	0.6	0.04
aVL_ston	0.6	-0.39
III_tptparea	0.6	-0.01
III_tarea	0.6	0
V4_rdur	0.59	0.19
V4_samp	0.59	-0.84
V6_tdur	0.59	0.81
V1_qamp	0.59	-0.03
V3_pppparea	0.59	0.08
V3_qrsppk	0.59	0.39
transttermmag	0.59	0.47
III_stend	0.59	-1.79
III_ramp	0.59	-0.05
V4_vat	0.59	0.04
aVL_qrsarea	0.59	0
V5_st80	0.59	0.11
aVF_sdur	0.59	-0.35
V4_pppparea	0.59	-0.03
qtintdispersion	0.59	0.05
III_st80	0.59	2.41
V5_tdur	0.58	0.43

V5_sdur	0.58	0.96
V1_print	0.58	0.04
V5_stmid	0.58	0.29
V5_pamp	0.58	-0.08
frontpinitmag	0.58	-0.2
V3_parea	0.58	0.05
II_sdur	0.58	-1.28
II_st80	0.58	-1.15
V2_qrsppk	0.58	0.23
V4_pamp	0.58	0
V4_parea	0.58	0.01
V1_stend	0.58	0.88
transptermangle	0.58	-0.04
frontptermangle	0.58	-0.03
V5_qrsppk	0.58	1.21
aVR_samp	0.58	-0.01
V4_tamp	0.58	0.03
transtmaxangle	0.58	-0.11
transttermangle	0.58	-0.01
V4_tptparea	0.58	0.08
V3_pamp	0.58	-0.1
II_stmid	0.58	-0.25

aVR_qdur	0.58	-0.48
V3_ston	0.58	0.38
V4_tarea	0.58	0.1
V5_parea	0.58	0.02
V2_parea	0.57	0.04
V5_pppparea	0.57	0.01
V2_pamp	0.57	-0.11
V2_qdur	0.57	-0.32
V2_qamp	0.57	0.07
aVR_rdur	0.57	-0.33
sagpcwrot	0.57	0.01
frontqrstermangle	0.57	-0.03
III_stmid	0.57	-0.07
III_sdur	0.57	-0.25
V1_st80	0.57	-0.23
avgpcount	0.57	0
transpinitmag	0.57	-0.13
sagqrstermangle	0.57	0.04
II_pamp	0.57	-0.04
aVF_tptparea	0.57	-0.19
transqrsinitmag	0.57	-0.4

aVL_samp	0.57	0.46
V4_tdur	0.57	-0.49
sagpinitangle	0.57	-0.04
frontpmaxmag	0.57	0.15
flutterfibcount	0.57	-0.03
aVF_tarea	0.57	-0.12
aVF_tamp	0.57	-0.11
atrialratestddev	0.57	0.03
t40frontaxis	0.57	-0.03
V1_ramp	0.57	0.19
V6_pamp	0.56	0
V2_qtint	0.56	0.09
V1_pppparea	0.56	-0.11
atrialrate	0.56	0.1
V1_stmid	0.56	-0.16
aVF_ston	0.56	0.48
V2_ppppdur	0.56	-0.03
V6_tptpdur	0.56	-0.86
V5_tptpdur	0.56	-0.42
V1_samp	0.56	-0.2
I_pppparea	0.56	-0.04
I_parea	0.56	-0.04

V6_parea	0.56	0.01
V2_ston	0.56	-0.02
II_prseg	0.56	0.01
qrsfrontaxis	0.56	0.08
highprint	0.56	0.2
frontpmaxangle	0.56	-0.01
V6_pppparea	0.56	0.01
sagqrscwrot	0.56	0.03
V3_stmid	0.56	-0.54
phorizaxis	0.56	0.01
V5_stslope	0.56	-0.2
transtcwrot	0.56	0.05
transpmaxangle	0.56	0.16
aVR_prseg	0.56	0.01
aVR_pamp	0.56	0.1
II_print	0.56	0.07
II_parea	0.56	0
sagttermmag	0.56	-0.1
II_pppparea	0.56	-0.02
frontpcwrot	0.56	-0.04
V3_qdur	0.56	-5.11

comppausecount	0.56	0.1
V3_qamp	0.56	0.27
aVR_qamp	0.56	-0.16
aVR_parea	0.56	-0.1
sagpmaxangle	0.56	0.06
V3_st80	0.56	-0.27
aVR_pppparea	0.56	-0.05
I_prseg	0.56	-0.04
aVF_samp	0.56	0.15
V1_pdur	0.55	0.04
V1_vat	0.55	-0.09
fronttcwrot	0.55	0.03
V5_rdur	0.55	0.6
V6_qrsppk	0.55	1.11
I_samp	0.55	0.19
III_qtint	0.55	-0.18
V5_samp	0.55	0.71
II_pdur	0.55	0.06
aVR_vat	0.55	0.01
V4_tptpdur	0.55	0.43
frontptermmag	0.55	-0.13
V3_qtint	0.55	0.1

transpmaxmag	0.55	0.29
II_samp	0.55	0.04
aVF_prseg	0.55	0.01
aVL_sdur	0.55	-0.47
V3_pdur	0.55	0.06
aVL_prseg	0.55	0.11
aVR_sdur	0.55	-0.24
sagpinitmag	0.55	0.23
aVF_tptpdur	0.55	0.3
III_print	0.55	-0.04
aVL_print	0.55	-0.1
I_pdur	0.55	0
aVF_print	0.55	-0.02
V6_qamp	0.55	0.16
qrshorizaxis	0.55	-0.01
meanqtint	0.55	0.83
aVF_pamp	0.55	0.16
frontpinitangle	0.55	0.05
V5_pdur	0.54	-0.1
aVR_pdur	0.54	0
aVF_stslope	0.54	-0.21
frontqrscwrot	0.54	-0.05

aVF_parea	0.54	-0.05
frontqrsinitmag	0.54	0.19
sagptermmag	0.54	0.29
V3_prseg	0.54	0.18
aVF_pppparea	0.54	-0.05
aVF_rdur	0.54	-0.28
V6_sdur	0.54	1.57
V3_stend	0.54	0.49
aVF_tdur	0.54	-0.32
aVL_qtint	0.54	0.08
V1_qrsppk	0.54	-0.17
V1_rdur	0.54	0.48
V1_qtint	0.54	0.06
aVR_print	0.54	0
V1_tdur	0.54	-0.2
transtmaxmag	0.54	-0.52
V6_prseg	0.54	-0.1
frontqrsinitangle	0.54	-0.1
V6_qdur	0.54	1.23
V1_rpamp	0.54	0.21
I_qamp	0.54	0.17
V1_rpdur	0.54	0.29

I_sdur	0.54	-0.65
V1_prseg	0.54	0.05
transpinitangle	0.54	0.09
aVR_ramp	0.54	0.1
aVR_rpamp	0.54	0.09
V1_tpamp	0.54	0.14
III_qrsppk	0.54	0.57
V6_pdur	0.54	-0.03
I_pamp	0.54	0.15
V1_tparea	0.54	-0.06
V3_tdur	0.54	-0.49
V1_stslope	0.53	-0.13
V1_tpdur	0.53	-0.1
V4_qtint	0.53	-0.06
t40horizaxis	0.53	-0.09
V4_pdur	0.53	-0.02
V3_print	0.53	-0.15
aVR_rpdur	0.53	-0.22
V5_vat	0.53	0.01
V1_parea	0.53	0
V2_print	0.53	0
V6_samp	0.53	0.38

V5_prseg	0.53	0.03
meanprint	0.53	-0.12
V2_samp	0.53	0.16
III_prseg	0.53	-0.01
V6_print	0.53	0.04
V5_print	0.53	-0.01
aVF_vat	0.53	0.04
I_print	0.53	0.02
II_stslope	0.53	-0.08
V1_pamp	0.53	0.05
III_samp	0.53	0.42
aVL_parea	0.53	-0.01
V4_qrsppk	0.53	-0.98
sagpmaxmag	0.53	-0.49
pfrontaxis	0.53	-0.04
aVL_pppparea	0.53	-0.03
V4_qdur	0.53	0.32
V2_stmid	0.53	0.08
V4_prseg	0.53	0
III_tdur	0.53	-0.06
aVF_pdur	0.53	-0.01
transqrsmaxmag	0.53	0.31

V4_qamp	0.53	-0.7
V3_tptpdur	0.53	0.42
printstddev	0.53	-0.13
V2_st80	0.53	0.07
V2_tdur	0.53	1.78
III_vat	0.52	0.11
aVL_qamp	0.52	0.53
III_qdur	0.52	-0.44
V4_print	0.52	0
III_ppppdur	0.52	-0.07
III_rpamp	0.52	0.01
V6_tpdur	0.52	0.59
aVF_stend	0.52	-1.27
V1_ppamp	0.52	0.25
V1_pparea	0.52	-0.31
III_rpdur	0.52	-0.36
V5_qtint	0.52	-0.01
aVF_qtint	0.52	0.07
III_pparea	0.52	0.07
III_tptpdur	0.52	0.09
aVL_rpdur	0.52	-0.23
aVL_rpamp	0.52	-0.17

V6_qrsarea	0.52	0.11
I_tpdur	0.52	1.88
I_ppppdur	0.52	0.03
aVL_tdur	0.52	0.62
I_rpdur	0.52	-0.2
I_rpamp	0.52	-0.02
III_ppamp	0.52	-0.1
V5_tpdur	0.52	0.24
aVL_pamp	0.52	0.01
aVR_qtint	0.52	0.17
I_qdur	0.52	-0.4
sagttermangle	0.52	0
aVL_tptpdur	0.52	-0.71
V3_stslope	0.52	-0.03
aVL_ppppdur	0.52	0.11
III_pamp	0.52	-0.13
III_qamp	0.52	0.19
meanprseg	0.52	-0.09
V2_rpdur	0.52	-0.41
V2_rpamp	0.52	0.07
III_ston	0.52	-0.31
III_rdur	0.52	-0.42

II_vat	0.52	0.03
V4_tpdur	0.52	-0.31
V2_tptpdur	0.52	-1.69
V6_ppppdur	0.52	0
V5_qamp	0.51	0.5
III_parea	0.51	0.03
V4_stend	0.51	-0.45
aVF_ppamp	0.51	-0.04
aVR_tpdur	0.51	0.94
III_pppparea	0.51	0.09
aVF_pparea	0.51	0.04
I_qrsarea	0.51	0.13
V1_tptpdur	0.51	0.02
II_qamp	0.51	0.08
V2_stend	0.51	-0.16
V3_ppppdur	0.51	0.01
V2_prseg	0.51	-0.07
notavgpbeats	0.51	-0.03
aVF_qdur	0.51	-0.19
V5_qdur	0.51	0.91
aVF_stmid	0.51	-0.75
V4_stmid	0.51	-0.16

V6_tpamp	0.51	-0.09
I_qtint	0.51	0.05
lowprint	0.51	0.04
V6_tparea	0.51	0.11
deltawavecount	0.51	0.04
II_tpdur	0.51	-0.15
V6_qtint	0.51	-0.04
V5_ppppdur	0.51	-0.01
aVF_st80	0.51	1.49
V2_tamp	0.51	0.16
aVR_ppppdur	0.51	0
V4_spamp	0.51	-0.06
V4_spdur	0.51	0.1
V5_rpdur	0.51	0.2
V5_rpamp	0.51	-0.11
V3_tamp	0.51	0.08
III_pdur	0.51	0.07
V4_rpamp	0.51	0.17
V4_ppppdur	0.51	0.04
aVR_tpamp	0.51	0.1
V4_rpdur	0.51	-0.01
transtinitmag	0.51	-0.21

II_tpamp	0.51	0.2
II_ppamp	0.51	0
transqrstermangle	0.51	0.04
V5_ppamp	0.51	0.06
V2_stslope	0.51	-0.08
aVL_pdur	0.51	-0.03
V5_spdur	0.51	0.11
V5_spamp	0.51	0.04
II_rpdur	0.51	-0.34
II_rpamp	0.51	0.06
aVF_qrsppk	0.51	0.6
II_spdur	0.51	-0.35
II_spamp	0.51	-0.07
II_tparea	0.51	-0.12
aVR_tparea	0.51	-0.01
V5_pparea	0.51	-0.07
aVL_spdur	0.51	-0.05
aVL_spamp	0.51	0.02
I_tpamp	0.51	-0.23
I_spdur	0.51	-0.02
II_qdur	0.51	-1.19
I_spamp	0.51	0.1

V4_stslope	0.51	-0.11
aVF_qamp	0.51	0.21
V3_tpdur	0.51	-0.32
V3_ppamp	0.51	0.03
V6_pparea	0.51	-0.03
I_tparea	0.51	0.17
deltawavepercent	0.51	-0.1
aVF_tpdur	0.51	-0.22
trigeminycount	0.51	0.23
trigeminystring	0.51	-0.24
V3_tarea	0.51	-0.09
III_tpdur	0.51	-0.07
aVF_ppppdur	0.51	0.03
aVF_rpamp	0.51	-0.15
V3_pparea	0.51	-0.07
II_pparea	0.51	-0.01
i40frontaxis	0.5	0.1
aVF_rpdur	0.5	-0.17
V6_ppamp	0.5	0
V2_tpdur	0.5	1.13
V4_st80	0.5	0.59
V6_rpamp	0.5	-0.24

V6_rpdur	0.5	0.37
III_spdur	0.5	-0.03
I_ppamp	0.5	-0.01
V4_tpamp	0.5	0.09
III_spamp	0.5	0
V5_tpamp	0.5	0.02
V3_tptparea	0.5	-0.1
aVL_qrsppk	0.5	0.61
V3_tparea	0.5	-0.21
bigeminycount	0.5	0.07
bigeminystring	0.5	-0.13
II_qtint	0.5	0.15
aVL_tpdur	0.5	0.35
V5_tparea	0.5	-0.1
V4_tparea	0.5	-0.09
aVL_pparea	0.5	0.07
V2_tparea	0.5	0.01
aVL_ppamp	0.5	-0.02
V1_spamp	0.5	-0.04
I_pparea	0.5	0.02
V1_spdur	0.5	0.05
aVR_pparea	0.5	-0.05

V3_tpamp	0.5	0.24
aVF_tparea	0.5	0.03
V2_spamp	0.5	0.05
V2_spdur	0.5	0.03
V2_ppamp	0.5	-0.12
V6_spdur	0.5	0.08
V6_spamp	0.5	-0.14
V2_tpamp	0.5	-0.02
aVL_tpamp	0.5	-0.02
aVF_tpamp	0.5	-0.04
aVL_tparea	0.5	-0.09
III_tparea	0.5	-0.08
V4_ston	0.5	0.15
V2_pdur	0.5	-0.03
III_tpamp	0.5	0.09
aVL_qdur	0.5	-0.2
aVL_ramp	0.5	-0.79
V4_pparea	0.5	0.03
V3_spamp	0.5	0.06
V3_spdur	0.5	-0.9
V2_pparea	0.5	0.09
aVR_spamp	0.5	0.05

aVR_spdur	0.5	-0.09
V1_sdur	0.5	0.5
aVF_spamp	0.5	-0.03
aVF_spdur	0.5	-0.14
aVR_ppamp	0.5	0.04
sagtmaxangle	0.5	-0.05
II_ppppdur	0.5	-0.03
V2_tarea	0.5	-0.14
V2_tptparea	0.5	-0.15
V3_rpdur	0.5	-2.3
wenckcount	0.5	-0.08
wenckstring	0.5	-0.31
V3_rpamp	0.5	-0.06
II_rdur	0.5	-1.13
V4_ppamp	0.5	-0.04

Supplemental Table 2

Performance of all models across three sites.

	Stanford	Stanford	Stanford	Stanford	Stanford
	AUC	Sensitivity @ 90%	Specificity @ 90%	Sensitivity @ equal specificity	PPV
Heart Rate	0.63 (0.61-0.65)	0.17 (0.15-0.19)	0.18 (0.17-0.18)	0.56 (0.53-0.59)	0.14 (0.13-0.15)
V3 Min. QRS Deflection	0.70 (0.68-0.72)	0.37 (0.34-0.39)	0.21 (0.20-0.21)	0.66 (0.63-0.69)	0.17 (0.16-0.18)
Mean QTc	0.75 (0.73-0.77)	0.39 (0.36-0.42)	0.30 (0.29-0.31)	0.69 (0.67-0.72)	0.20 (0.19-0.21)
V3 QRS Duration	0.75 (0.73-0.76)	0.36 (0.33-0.39)	0.29 (0.28-0.30)	0.69 (0.66-0.72)	0.20 (0.19-0.21)
aVR T Amplitude	0.77 (0.75-0.78)	0.34 (0.31-0.36)	0.43 (0.42-0.44)	0.72 (0.69-0.74)	0.21 (0.20-0.23)
5 Variable LR	0.86 (0.85-0.87)	0.57 (0.55-0.60)	0.66 (0.65-0.67)	0.79 (0.76-0.81)	0.28 (0.27-0.30)
5 Variable XGBoost	0.88 (0.87-0.89)	0.59 (0.56-0.62)	0.69 (0.68-0.70)	0.80 (0.77-0.82)	0.29 (0.28-0.31)
555 Variable LR	0.90 (0.89-0.91)	0.70 (0.68-0.73)	0.72 (0.71-0.73)	0.82 (0.80-0.84)	0.33 (0.31-0.35)
555 Variable XGBoost	0.92 (0.91-0.93)	0.76 (0.73-0.78)	0.79 (0.78-0.80)	0.84 (0.82-0.86)	0.37 (0.35-0.39)
AI-ECG	0.94 (0.93-0.94)	0.80 (0.78-0.83)	0.82 (0.81-0.83)	0.86 (0.84-0.88)	0.40 (0.38-0.42)

	UKB	UKB	UKB	UKB	UKB
	AUC	Sensitivity @ 90%	Specificity @ 90%	Sensitivity @ equal specificity	PPV
Heart Rate	0.67 (0.61-0.74)	0.42 (0.32-0.52)	0.15 (0.15-0.15)	0.64 (0.54-0.73)	0.00 (0.00-0.01)
V3 Min. QRS Deflection	0.59 (0.52-0.65)	0.26 (0.17-0.35)	0.12 (0.11-0.12)	0.55 (0.45-0.65)	0.00 (0.00-0.00)
Mean QTc	0.68 (0.62-0.75)	0.38 (0.28-0.48)	0.13 (0.12-0.13)	0.64 (0.54-0.73)	0.00 (0.00-0.01)
V3 QRS Duration	0.68 (0.62-0.74)	0.38 (0.27-0.47)	0.16 (0.16-0.17)	0.62 (0.52-0.72)	0.00 (0.00-0.01)
aVR T Amplitude	0.78 (0.73-0.83)	0.49 (0.40-0.60)	0.38 (0.38-0.39)	0.71 (0.62-0.81)	0.01 (0.00-0.01)
5 Variable LR	0.83 (0.78-0.88)	0.60 (0.51-0.70)	0.48 (0.47-0.48)	0.73 (0.62-0.82)	0.01 (0.01-0.01)
5 Variable XGBoost	0.82 (0.77-0.87)	0.57 (0.47-0.67)	0.44 (0.43-0.44)	0.77 (0.68-0.85)	0.01 (0.01-0.01)
555 Variable LR					
555 Variable XGBoost					
AI-ECG	0.72 (0.67-0.77)	0.34 (0.25-0.45)	0.36 (0.35-0.36)	0.66 (0.56-0.75)	0.01 (0.00-0.01)

	Columbia	Columbia	Columbia	Columbia	Columbia
	AUC	Sensitivity @ 90%	Specificity @ 90%	Sensitivity @ equal specificity	PPV
Heart Rate	0.57 (0.57-0.58)	0.11 (0.10-0.12)	0.09 (0.09-0.09)	0.53 (0.52-0.55)	0.15 (0.15-0.16)
V3 Min. QRS Deflection	0.64 (0.63-0.65)	0.29 (0.28-0.30)	0.13 (0.12-0.13)	0.61 (0.59-0.62)	0.18 (0.17-0.19)
Mean QTc	0.72 (0.71-0.73)	0.35 (0.34-0.37)	0.26 (0.25-0.26)	0.66 (0.65-0.68)	0.22 (0.22-0.23)
V3 QRS Duration	0.64 (0.63-0.65)	0.27 (0.26-0.28)	0.18 (0.18-0.19)	0.56 (0.55-0.58)	0.18 (0.17-0.18)
aVR T Amplitude	0.74 (0.74-0.75)	0.28 (0.27-0.29)	0.39 (0.38-0.39)	0.69 (0.67-0.70)	0.25 (0.24-0.26)
5 Variable LR	0.80 (0.80-0.81)	0.44 (0.42-0.45)	0.49 (0.49-0.50)	0.73 (0.72-0.74)	0.28 (0.27-0.29)
5 Variable XGBoost	0.81 (0.80-0.82)	0.41 (0.40-0.43)	0.55 (0.54-0.55)	0.73 (0.72-0.75)	0.28 (0.28-0.29)
555 Variable LR					
555 Variable XGBoost					
AI-ECG	0.88 (0.87-0.88)	0.65 (0.63-0.66)	0.66 (0.66-0.67)	0.80 (0.79-0.81)	0.37 (0.36-0.38)

Supplemental Figures

Supplemental Figure 1

Performance of several risk scores in detecting LVSD, by AUROC (area under receiver operator characteristic), specificity at a cutoff providing 90% sensitivity, sensitivity at a cutoff providing 90% sensitivity, sensitivity at a cutoff balancing sensitivity and specificity, and positive predictive value. Error bars are 95% bootstrap confidence intervals.

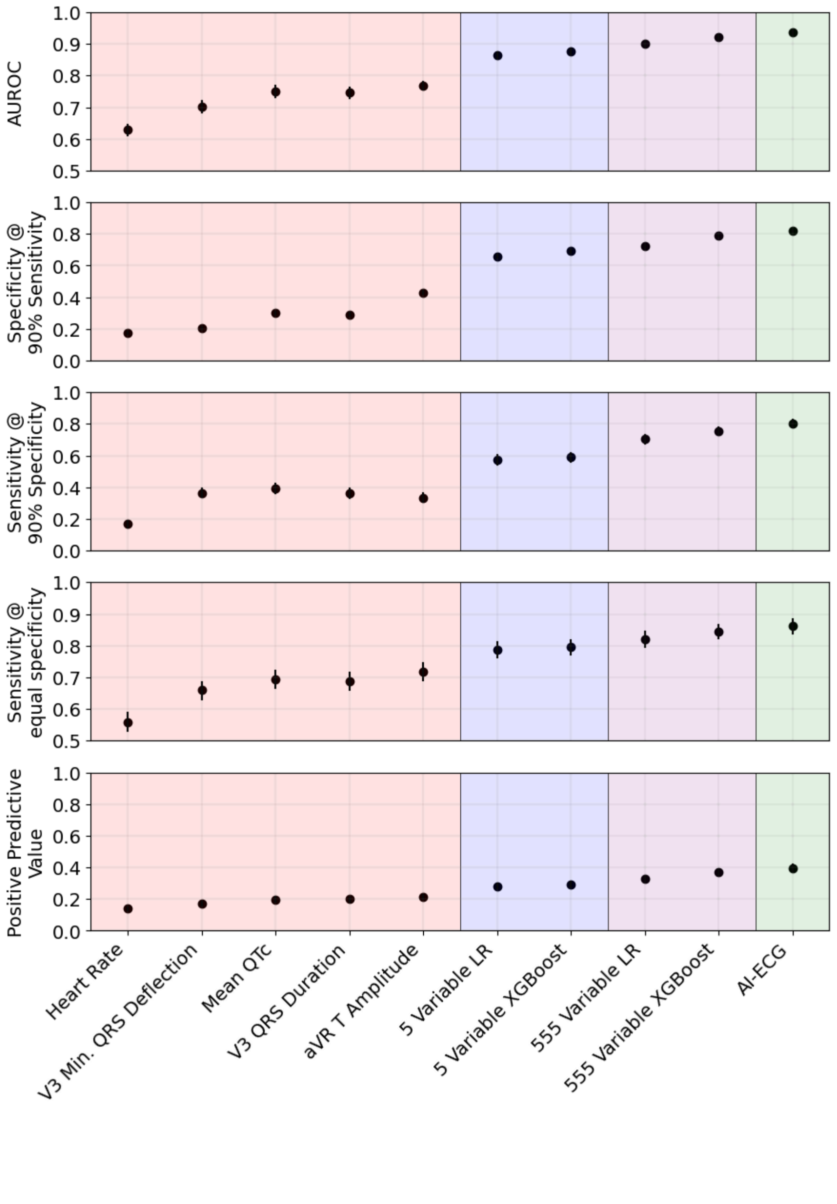

Supplemental Figure 2

Performance of linear models built on 1 to 8 measurements on the Stanford test set. On the X axis, each tick represents the addition of another variable, i.e. the first tick is a model based only on the aVR T-wave amplitude, the second based on the aVR T-wave amplitude and V3 QRS duration, and so on.

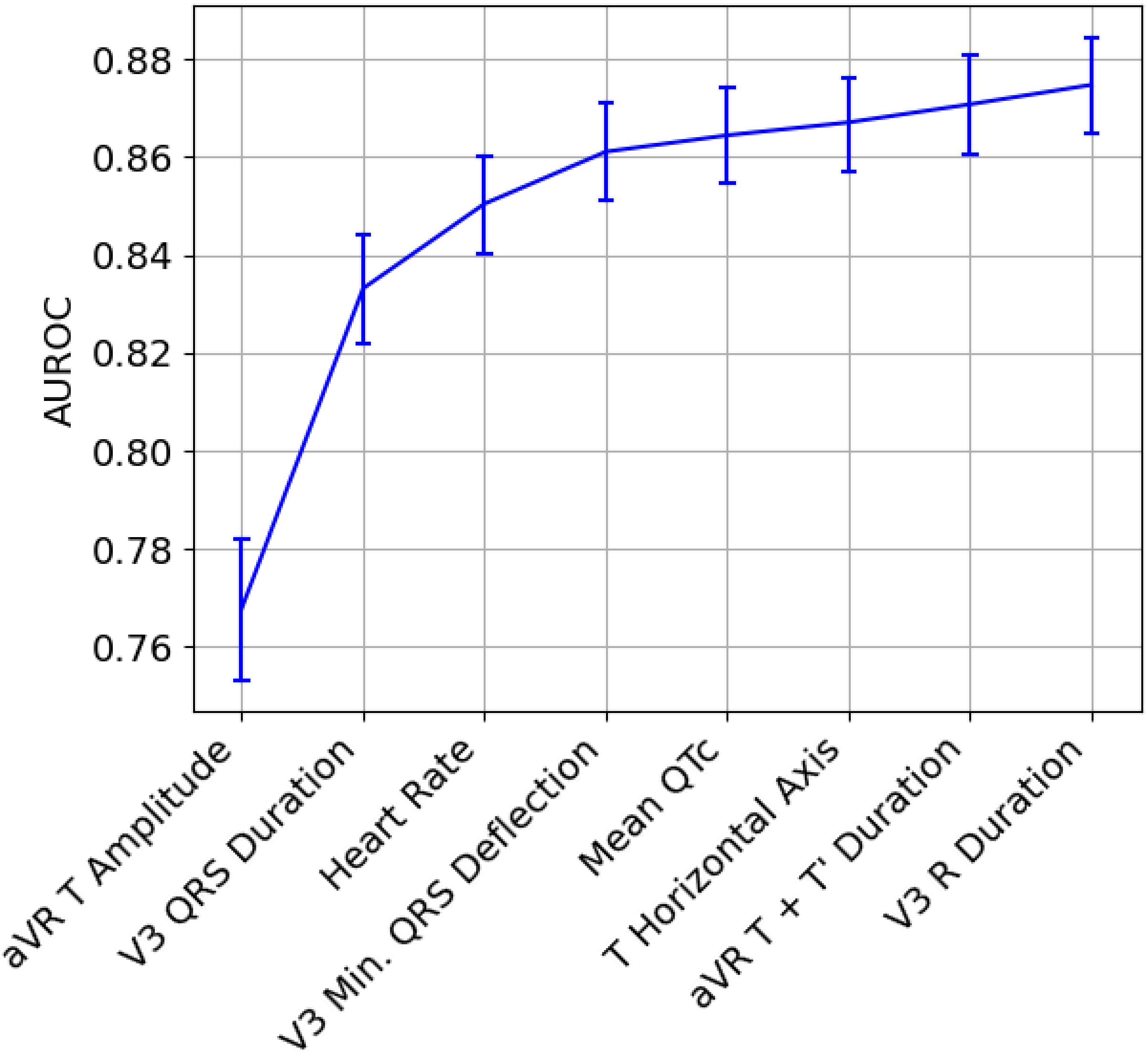

Supplement: ztae034_Supplementary_Data [file ztae034_supplementary_data.pdf]
